# 3α-Hydr­oxy-*N*-(3-hydroxy­prop­yl)-5β-cholan-24-amide

**DOI:** 10.1107/S1600536809006862

**Published:** 2009-02-28

**Authors:** Arto Valkonen, Juha Koivukorpi, Manu Lahtinen, Erkki Kolehmainen

**Affiliations:** a University of Jyväskylä, Department of Chemistry, PO Box 35, FIN-40014 Jyväskylä, Finland

## Abstract

The title compound, C_27_H_47_NO_3_, is a (3-hydroxy­prop­yl)amide derivative of naturally occurring enanti­opure lithocholic acid (3α-hydr­oxy-5β-cholan-24-oic acid). The mol­ecule contains four fused rings: three six-membered rings in chair conformations and one five-membered ring in a half-chair form. The two terminal six-membered rings are *cis*-fused, while other rings are *trans*-fused. The structure contains an intra­molecular O—H⋯O hydrogen bond and a similar hydrogen-bond framework to the corresponding deoxy­cholic and chenodeoxy­cholic acid derivatives. Inter­molecular O—H⋯O and N—H⋯O inter­actions are also present in the crystal. This compound seems to have at least two polymorphic forms from a comparison of the X-ray powder pattern simulated from the present structure of the title compound and that previously obtained for the powder sample.

## Related literature

For general background, see: Tamminen *et al.* (2000 ▶); Valkonen *et al.* (2004 ▶); Valkonen (2008 ▶). For related structures, see: Valkonen *et al.* (2007 ▶, 2008 ▶).
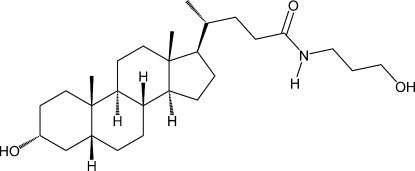


## Experimental

### 

#### Crystal data

C_27_H_47_NO_3_
*M* *_r_* = 433.66
Monoclinic, 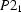

*a* = 11.4462 (5) Å
*b* = 7.5998 (3) Å
*c* = 14.3286 (6) Å
β = 102.055 (2)°
*V* = 1218.94 (9) Å^3^
*Z* = 2
Mo *K*α radiation
μ = 0.08 mm^−1^
*T* = 123 K
0.30 × 0.10 × 0.06 mm

#### Data collection

Bruker Kappa APEXII diffractometer
Absorption correction: none
9113 measured reflections
3155 independent reflections
2207 reflections with *I* > 2σ(*I*)
*R*_int_ = 0.091

#### Refinement

*R*[*F* ^2^ > 2σ(*F* ^2^)] = 0.067
*wR*(*F* ^2^) = 0.135
*S* = 1.05
3155 reflections
289 parameters
4 restraints
H atoms treated by a mixture of independent and constrained refinement
Δρ_max_ = 0.32 e Å^−3^
Δρ_min_ = −0.32 e Å^−3^

### 

Data collection: *COLLECT* (Bruker, 2008 ▶); cell refinement: *DENZO-SMN* (Otwinowski & Minor, 1997 ▶); data reduction: *DENZO-SMN*; program(s) used to solve structure: *SIR2002* (Burla *et al.*, 2003 ▶); program(s) used to refine structure: *SHELXL97* (Sheldrick, 2008 ▶); molecular graphics: *ORTEP-3* (Farrugia, 1997 ▶); software used to prepare material for publication: *SHELXL97* and *Mercury* (Macrae *et al.*, 2006 ▶).

## Supplementary Material

Crystal structure: contains datablocks global, I. DOI: 10.1107/S1600536809006862/is2393sup1.cif

Structure factors: contains datablocks I. DOI: 10.1107/S1600536809006862/is2393Isup2.hkl

Additional supplementary materials:  crystallographic information; 3D view; checkCIF report

## Figures and Tables

**Table 1:** Hydrogen-bond geometry (Å, °)

*D*—H⋯*A*	*D*—H	H⋯*A*	*D*⋯*A*	*D*—H⋯*A*
O27—H27*O*⋯O24	0.86 (4)	1.98 (2)	2.810 (4)	164 (5)
O3—H3*O*⋯O24^i^	0.84 (2)	2.05 (2)	2.880 (5)	171 (5)
N24—H24⋯O3^i^	0.89 (2)	2.20 (3)	3.032 (5)	155 (4)

Symmetry code: (i) 
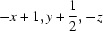
.

**Fig. 1.**
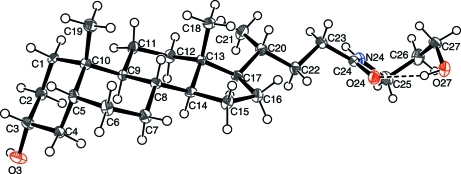
View of the molecule of (I) showing the atom-labeling scheme. Displacement ellipsoids are drawn at the 50% probability level. H atoms are represented by circles of arbitrary size.

**Fig. 2.**
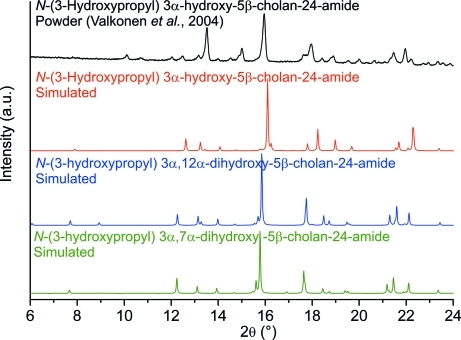
The experimental powder diffraction pattern of powdery sample and the simulated pattern from the single-crystal structure of title compound. The simulated patterns of analogous DCA and CDCA derivatives are also presented for comparison.

## supplementary crystallographic information

### Comment

The title compound is a lithocholic acid (LCA) derivative which was supposed to be a potential organogelating agent (Valkonen *et al.*, 2004). However, in gelation studies these properties were found to be too weak for utilization in any purposes. Although single crystals of analogous deoxycholic (DCA, 3α,12α-dihydroxy-5β-cholan-24-oic acid) and chenodeoxycholic (CDCA, 3α,7α-dihydroxy-5β-cholan-24-oic acid) acid amide derivatives were easily obtained during gelation tests (Valkonen *et al.*, 2004; Valkonen *et al.*, 2007; Valkonen *et al.*, 2008), the crystals of the title compound were very thin needles and far too small for crystallographic data collection. Methanol, which is unacceptably good solvent for the title compound and analogues in gel formation (Valkonen *et al.*, 2004), showed to be a good solvent for growing of reasonable size crystals of the title compound for X-ray diffraction studies. The molecular structure of the title compound is shown in Fig. 1.

The simulated powder diffraction pattern by Mercury (Macrae *et al.*, 2006) from the single crystals of title compound in Fig. 2 is not congruent with the powdery sample pattern previously investigated (Valkonen *et al.*, 2004), indicating the title compound to have more than one polymorphic form. However, the single-crystal structure of title compound is isostructural to analogous DCA and CDCA derivatives, *N*-(3-hydroxypropyl) 3α,12α-dihydroxy-5β-cholan-24-amide and *N*-(3-hydroxypropyl) 3α,7α-dihydroxy-5β-cholan-24-amide, as also seen from the simulated powder diffraction patterns in Fig. 2. These compounds have also similar unit-cell parameters, an intramolecular O—H···O hydrogen bond between hydroxyl group (O27—H27o) at the end of the side chain and amide carbonyl (O24) (Fig. 1 and Table 1) as well as similar *ttt*i side chain overall conformation (Valkonen *et al.*, 2008; Valkonen, 2008). The intermolecular H-bond frameworks are also identical, which is possible due to the lack of the acceptors for the extra O—H donors in structures of DCA and CDCA derivatives.

### Experimental

The first step was a preparation of methyl lithocholate from lithocholic acid according to literature method (Tamminen *et al.*, 2000). In the second step methyl lithocholate (1.69 g, 4.33 mmol) and 3-amino-1-propanol (3.25 g, 43.3 mmol) were dissolved in 20 ml of methanol. The resulting mixture was heated with an oil bath and stirred at 70–80 °C for 2 days. Cooled solution was poured into 50 ml of water, the precipitate was filtered and washed twice with water. The obtained product was dried and recrystallized from acetonitrile. Yield was 1.48 g (79%).

Suitable single crystals for X-ray diffraction were obtained by very slow evaporation of analytical sample from NMR-tube, where methanol-d_4_ was used as a solvent. The melting point of these single crystals (186–188 °C) was found to be in agreement with the one for powdery product (184–185 °C, Valkonen *et al.*, 2004).

### Refinement

In the absence of significant anomalous scattering effects Friedel pairs have been merged. The meaningless Flack parameter is not reported. All H atoms were visible in electron density maps, but those bonded to C were placed at idealized positions and allowed to ride on their parent atoms at C—H distances of 0.98 Å (methyl), 0.99 Å (methylene), and 1.00 Å (methine), with *U*_iso_(H) of 1.2 times *U*_eq_(C) (or 1.5 times *U*_eq_(C) for methyls). The N—H proton was found in the electron density map and it was fixed in place by *DFIX* restraint at distance of 0.91 (2) Å from N atom, and *U*_iso_(H) value of 1.2 times *U*_eq_(N) was used. The O—H protons were also found in the electron density map, restrained by *DFIX* [0.84 (2) Å from O] and *U*_iso_(H) factors set to values of 1.5 times *U*_eq_(O).

### Crystal data

**Table:**

C_27_H_47_NO_3_	*F*(000) = 480
*M**_r_* = 433.66	*D*_x_ = 1.182 Mg m^−^^3^
Monoclinic, *P*2_1_	Melting point = 459–461 K
Hall symbol: P 2yb	Mo *K*α radiation, λ = 0.71073 Å
*a* = 11.4462 (5) Å	Cell parameters from 4982 reflections
*b* = 7.5998 (3) Å	θ = 0.4–28.3°
*c* = 14.3286 (6) Å	µ = 0.08 mm^−^^1^
β = 102.055 (2)°	*T* = 123 K
*V* = 1218.94 (9) Å^3^	Block, colourless
*Z* = 2	0.30 × 0.10 × 0.06 mm

### Data collection

**Table:**

Bruker Kappa APEXII diffractometer	2207 reflections with *I* > 2σ(*I*)
Radiation source: fine-focus sealed tube	*R*_int_ = 0.091
graphite	θ_max_ = 28.0°, θ_min_ = 2.1°
Detector resolution: 9 pixels mm^-1^	*h* = −15→15
φ and ω scans	*k* = −8→10
9113 measured reflections	*l* = −16→18
3155 independent reflections	

### Refinement

**Table:**

Refinement on *F*^2^	Primary atom site location: structure-invariant direct methods
Least-squares matrix: full	Secondary atom site location: difference Fourier map
*R*[*F*^2^ > 2σ(*F*^2^)] = 0.067	Hydrogen site location: inferred from neighbouring sites
*wR*(*F*^2^) = 0.135	H atoms treated by a mixture of independent and constrained refinement
*S* = 1.05	*w* = 1/[σ^2^(*F*_o_^2^) + (0.0324*P*)^2^ + 0.9968*P*] where *P* = (*F*_o_^2^ + 2*F*_c_^2^)/3
3155 reflections	(Δ/σ)_max_ < 0.001
289 parameters	Δρ_max_ = 0.32 e Å^−^^3^
4 restraints	Δρ_min_ = −0.32 e Å^−^^3^

### Special details

**Table:**

Geometry. All e.s.d.'s (except the e.s.d. in the dihedral angle between two l.s. planes) are estimated using the full covariance matrix. The cell e.s.d.'s are taken into account individually in the estimation of e.s.d.'s in distances, angles and torsion angles; correlations between e.s.d.'s in cell parameters are only used when they are defined by crystal symmetry. An approximate (isotropic) treatment of cell e.s.d.'s is used for estimating e.s.d.'s involving l.s. planes.
Refinement. Refinement of *F*^2^ against ALL reflections. The weighted *R*-factor *wR* and goodness of fit *S* are based on *F*^2^, conventional *R*-factors *R* are based on *F*, with *F* set to zero for negative *F*^2^. The threshold expression of *F*^2^ > σ(*F*^2^) is used only for calculating *R*-factors(gt) *etc*. and is not relevant to the choice of reflections for refinement. *R*-factors based on *F*^2^ are statistically about twice as large as those based on *F*, and *R*- factors based on ALL data will be even larger.

### Fractional atomic coordinates and isotropic or equivalent isotropic displacement parameters (Å^2^)

**Table:**

	*x*	*y*	*z*	*U*_iso_*/*U*_eq_	
O3	0.1237 (2)	0.3301 (5)	0.2779 (2)	0.0286 (7)	
H3O	0.098 (4)	0.433 (4)	0.267 (4)	0.043*	
O24	0.9728 (3)	0.1814 (4)	−0.2630 (2)	0.0254 (7)	
O27	1.1324 (3)	0.1259 (5)	−0.3848 (2)	0.0321 (8)	
H27O	1.076 (3)	0.128 (8)	−0.354 (3)	0.048*	
N24	0.9619 (3)	0.4638 (5)	−0.3142 (2)	0.0228 (8)	
H24	0.961 (4)	0.576 (3)	−0.296 (3)	0.027*	
C1	0.4412 (3)	0.4854 (6)	0.3733 (3)	0.0174 (9)	
H1A	0.4387	0.4852	0.4419	0.021*	
H1B	0.4853	0.5920	0.3608	0.021*	
C2	0.3124 (4)	0.4976 (6)	0.3147 (3)	0.0194 (9)	
H2A	0.2734	0.6048	0.3329	0.023*	
H2B	0.3132	0.5051	0.2459	0.023*	
C3	0.2436 (3)	0.3357 (6)	0.3338 (3)	0.0208 (8)	
H3	0.2402	0.3336	0.4030	0.025*	
C4	0.3066 (4)	0.1691 (5)	0.3105 (3)	0.0171 (9)	
H4A	0.2625	0.0651	0.3264	0.021*	
H4B	0.3044	0.1660	0.2411	0.021*	
C5	0.4362 (4)	0.1573 (6)	0.3644 (3)	0.0179 (9)	
H5	0.4350	0.1483	0.4338	0.021*	
C6	0.4926 (4)	−0.0133 (6)	0.3366 (3)	0.0209 (9)	
H6A	0.5668	−0.0375	0.3843	0.025*	
H6B	0.4368	−0.1121	0.3386	0.025*	
C7	0.5222 (4)	−0.0058 (6)	0.2371 (3)	0.0197 (9)	
H7A	0.5658	−0.1139	0.2264	0.024*	
H7B	0.4471	−0.0016	0.1883	0.024*	
C8	0.5982 (4)	0.1547 (5)	0.2259 (3)	0.0150 (8)	
H8	0.6760	0.1446	0.2729	0.018*	
C9	0.5346 (3)	0.3249 (6)	0.2484 (2)	0.0149 (7)	
H9	0.4543	0.3255	0.2044	0.018*	
C10	0.5113 (3)	0.3215 (6)	0.3519 (3)	0.0162 (8)	
C11	0.5986 (4)	0.4930 (6)	0.2267 (3)	0.0193 (9)	
H11A	0.6721	0.5084	0.2765	0.023*	
H11B	0.5461	0.5952	0.2303	0.023*	
C12	0.6329 (4)	0.4923 (6)	0.1274 (3)	0.0205 (9)	
H12A	0.5593	0.4972	0.0769	0.025*	
H12B	0.6810	0.5983	0.1213	0.025*	
C13	0.7040 (3)	0.3283 (6)	0.1135 (2)	0.0150 (7)	
C14	0.6243 (4)	0.1675 (5)	0.1261 (3)	0.0153 (9)	
H14	0.5458	0.1856	0.0812	0.018*	
C15	0.6835 (4)	0.0112 (5)	0.0885 (3)	0.0210 (9)	
H15A	0.6244	−0.0823	0.0652	0.025*	
H15B	0.7478	−0.0382	0.1388	0.025*	
C16	0.7354 (4)	0.0897 (6)	0.0050 (3)	0.0198 (9)	
H16A	0.8204	0.0563	0.0120	0.024*	
H16B	0.6904	0.0452	−0.0571	0.024*	
C17	0.7229 (3)	0.2934 (5)	0.0104 (3)	0.0161 (9)	
H17	0.6474	0.3272	−0.0349	0.019*	
C18	0.8249 (3)	0.3242 (7)	0.1850 (3)	0.0213 (8)	
H18A	0.8689	0.2179	0.1745	0.032*	
H18B	0.8112	0.3237	0.2502	0.032*	
H18C	0.8717	0.4284	0.1757	0.032*	
C19	0.6294 (3)	0.3191 (7)	0.4273 (3)	0.0219 (8)	
H19A	0.6765	0.4238	0.4196	0.033*	
H19B	0.6749	0.2132	0.4189	0.033*	
H19C	0.6117	0.3188	0.4914	0.033*	
C20	0.8261 (4)	0.3916 (6)	−0.0206 (3)	0.0201 (9)	
H20	0.9017	0.3593	0.0250	0.024*	
C21	0.8120 (5)	0.5929 (6)	−0.0172 (3)	0.0309 (11)	
H21A	0.8052	0.6285	0.0472	0.046*	
H21B	0.7400	0.6286	−0.0631	0.046*	
H21C	0.8820	0.6495	−0.0336	0.046*	
C22	0.8381 (3)	0.3326 (7)	−0.1215 (3)	0.0200 (8)	
H22A	0.8413	0.2025	−0.1234	0.024*	
H22B	0.7663	0.3710	−0.1684	0.024*	
C23	0.9502 (4)	0.4082 (6)	−0.1506 (3)	0.0221 (9)	
H23A	0.9454	0.5382	−0.1518	0.027*	
H23B	1.0219	0.3740	−0.1025	0.027*	
C24	0.9626 (3)	0.3426 (6)	−0.2473 (3)	0.0198 (8)	
C25	0.9603 (4)	0.4214 (6)	−0.4139 (3)	0.0265 (10)	
H25A	0.9074	0.5057	−0.4555	0.032*	
H25B	0.9265	0.3021	−0.4280	0.032*	
C26	1.0851 (4)	0.4280 (6)	−0.4374 (3)	0.0312 (11)	
H26A	1.0780	0.3998	−0.5058	0.037*	
H26B	1.1165	0.5493	−0.4269	0.037*	
C27	1.1732 (4)	0.3029 (7)	−0.3787 (3)	0.0268 (11)	
H27A	1.2496	0.3091	−0.4006	0.032*	
H27B	1.1888	0.3408	−0.3111	0.032*	

### Atomic displacement parameters (Å^2^)

**Table:**

	*U*^11^	*U*^22^	*U*^33^	*U*^12^	*U*^13^	*U*^23^
O3	0.0175 (14)	0.0220 (15)	0.0440 (18)	−0.0023 (16)	0.0010 (12)	−0.0018 (17)
O24	0.0259 (16)	0.0213 (17)	0.0316 (17)	−0.0016 (15)	0.0119 (13)	−0.0033 (15)
O27	0.0307 (18)	0.037 (2)	0.0309 (18)	0.0040 (16)	0.0128 (14)	−0.0078 (15)
N24	0.0225 (18)	0.025 (2)	0.0227 (18)	0.0037 (17)	0.0092 (15)	0.0037 (17)
C1	0.018 (2)	0.020 (2)	0.0136 (19)	−0.0009 (19)	0.0023 (16)	−0.0057 (17)
C2	0.020 (2)	0.016 (2)	0.023 (2)	0.0025 (19)	0.0064 (17)	−0.0003 (19)
C3	0.0142 (17)	0.023 (2)	0.025 (2)	0.001 (2)	0.0027 (15)	0.001 (2)
C4	0.018 (2)	0.012 (2)	0.024 (2)	−0.0014 (18)	0.0083 (16)	−0.0031 (18)
C5	0.020 (2)	0.017 (2)	0.019 (2)	0.0024 (19)	0.0081 (17)	0.0027 (17)
C6	0.024 (2)	0.014 (2)	0.027 (2)	0.0026 (19)	0.0114 (17)	0.0054 (18)
C7	0.020 (2)	0.015 (2)	0.025 (2)	0.0020 (19)	0.0076 (17)	0.0019 (18)
C8	0.015 (2)	0.012 (2)	0.018 (2)	0.0003 (18)	0.0032 (16)	0.0004 (17)
C9	0.0143 (17)	0.0155 (18)	0.0154 (18)	0.000 (2)	0.0040 (14)	−0.0013 (19)
C10	0.0186 (18)	0.0147 (18)	0.0159 (18)	0.000 (2)	0.0047 (15)	0.0007 (18)
C11	0.026 (2)	0.012 (2)	0.024 (2)	−0.003 (2)	0.0116 (18)	−0.0034 (19)
C12	0.027 (2)	0.018 (2)	0.018 (2)	−0.001 (2)	0.0094 (18)	0.0033 (18)
C13	0.0155 (17)	0.0169 (18)	0.0135 (17)	−0.001 (2)	0.0052 (14)	−0.0002 (18)
C14	0.018 (2)	0.009 (2)	0.018 (2)	−0.0027 (18)	0.0027 (16)	0.0016 (17)
C15	0.031 (2)	0.014 (2)	0.020 (2)	−0.0002 (19)	0.0091 (18)	0.0006 (17)
C16	0.024 (2)	0.016 (2)	0.021 (2)	0.0014 (18)	0.0076 (18)	0.0016 (17)
C17	0.0156 (18)	0.019 (2)	0.0130 (18)	−0.0013 (18)	0.0007 (14)	0.0009 (16)
C18	0.0200 (18)	0.024 (2)	0.0191 (19)	−0.003 (2)	0.0031 (15)	−0.003 (2)
C19	0.0180 (18)	0.025 (2)	0.022 (2)	0.003 (2)	0.0018 (15)	0.001 (2)
C20	0.023 (2)	0.023 (2)	0.016 (2)	−0.0030 (18)	0.0072 (17)	0.0012 (16)
C21	0.044 (3)	0.023 (2)	0.031 (3)	−0.013 (2)	0.022 (2)	−0.007 (2)
C22	0.0204 (18)	0.021 (2)	0.0191 (19)	0.004 (2)	0.0053 (15)	0.0011 (19)
C23	0.019 (2)	0.026 (2)	0.023 (2)	−0.0045 (19)	0.0084 (18)	−0.0023 (18)
C24	0.0152 (18)	0.024 (2)	0.021 (2)	−0.001 (2)	0.0050 (15)	−0.004 (2)
C25	0.022 (2)	0.032 (3)	0.024 (2)	0.008 (2)	0.0022 (18)	0.0090 (19)
C26	0.039 (3)	0.034 (3)	0.025 (2)	0.003 (2)	0.015 (2)	0.004 (2)
C27	0.0182 (19)	0.042 (3)	0.021 (2)	−0.005 (2)	0.0055 (16)	−0.004 (2)

### Geometric parameters (Å, °)

**Table:**

O3—C3	1.438 (4)	C12—H12B	0.9900
O3—H3O	0.84 (2)	C13—C18	1.541 (5)
O24—C24	1.256 (5)	C13—C14	1.558 (6)
O27—C27	1.420 (6)	C13—C17	1.559 (5)
O27—H27O	0.86 (4)	C14—C15	1.520 (6)
N24—C24	1.328 (6)	C14—H14	1.0000
N24—C25	1.460 (5)	C15—C16	1.561 (5)
N24—H24	0.89 (2)	C15—H15A	0.9900
C1—C2	1.539 (5)	C15—H15B	0.9900
C1—C10	1.546 (6)	C16—C17	1.558 (6)
C1—H1A	0.9900	C16—H16A	0.9900
C1—H1B	0.9900	C16—H16B	0.9900
C2—C3	1.516 (6)	C17—C20	1.539 (5)
C2—H2A	0.9900	C17—H17	1.0000
C2—H2B	0.9900	C18—H18A	0.9800
C3—C4	1.528 (6)	C18—H18B	0.9800
C3—H3	1.0000	C18—H18C	0.9800
C4—C5	1.525 (5)	C19—H19A	0.9800
C4—H4A	0.9900	C19—H19B	0.9800
C4—H4B	0.9900	C19—H19C	0.9800
C5—C6	1.537 (6)	C20—C21	1.540 (6)
C5—C10	1.546 (6)	C20—C22	1.547 (5)
C5—H5	1.0000	C20—H20	1.0000
C6—C7	1.534 (5)	C21—H21A	0.9800
C6—H6A	0.9900	C21—H21B	0.9800
C6—H6B	0.9900	C21—H21C	0.9800
C7—C8	1.526 (5)	C22—C23	1.541 (5)
C7—H7A	0.9900	C22—H22A	0.9900
C7—H7B	0.9900	C22—H22B	0.9900
C8—C14	1.524 (5)	C23—C24	1.507 (5)
C8—C9	1.551 (5)	C23—H23A	0.9900
C8—H8	1.0000	C23—H23B	0.9900
C9—C11	1.537 (6)	C25—C26	1.535 (6)
C9—C10	1.562 (5)	C25—H25A	0.9900
C9—H9	1.0000	C25—H25B	0.9900
C10—C19	1.544 (5)	C26—C27	1.508 (6)
C11—C12	1.553 (5)	C26—H26A	0.9900
C11—H11A	0.9900	C26—H26B	0.9900
C11—H11B	0.9900	C27—H27A	0.9900
C12—C13	1.524 (6)	C27—H27B	0.9900
C12—H12A	0.9900		
			
C3—O3—H3O	109 (4)	C14—C13—C17	100.2 (3)
C27—O27—H27O	103 (4)	C15—C14—C8	118.1 (3)
C24—N24—C25	123.4 (4)	C15—C14—C13	104.9 (3)
C24—N24—H24	117 (3)	C8—C14—C13	113.2 (3)
C25—N24—H24	120 (3)	C15—C14—H14	106.7
C2—C1—C10	114.7 (3)	C8—C14—H14	106.7
C2—C1—H1A	108.6	C13—C14—H14	106.7
C10—C1—H1A	108.6	C14—C15—C16	104.0 (3)
C2—C1—H1B	108.6	C14—C15—H15A	111.0
C10—C1—H1B	108.6	C16—C15—H15A	111.0
H1A—C1—H1B	107.6	C14—C15—H15B	111.0
C3—C2—C1	109.1 (3)	C16—C15—H15B	111.0
C3—C2—H2A	109.9	H15A—C15—H15B	109.0
C1—C2—H2A	109.9	C17—C16—C15	106.7 (3)
C3—C2—H2B	109.9	C17—C16—H16A	110.4
C1—C2—H2B	109.9	C15—C16—H16A	110.4
H2A—C2—H2B	108.3	C17—C16—H16B	110.4
O3—C3—C2	113.2 (3)	C15—C16—H16B	110.4
O3—C3—C4	107.0 (3)	H16A—C16—H16B	108.6
C2—C3—C4	110.3 (3)	C20—C17—C16	112.6 (3)
O3—C3—H3	108.7	C20—C17—C13	117.2 (3)
C2—C3—H3	108.7	C16—C17—C13	104.4 (3)
C4—C3—H3	108.7	C20—C17—H17	107.4
C5—C4—C3	113.1 (3)	C16—C17—H17	107.4
C5—C4—H4A	109.0	C13—C17—H17	107.4
C3—C4—H4A	109.0	C13—C18—H18A	109.5
C5—C4—H4B	109.0	C13—C18—H18B	109.5
C3—C4—H4B	109.0	H18A—C18—H18B	109.5
H4A—C4—H4B	107.8	C13—C18—H18C	109.5
C4—C5—C6	109.6 (3)	H18A—C18—H18C	109.5
C4—C5—C10	113.5 (3)	H18B—C18—H18C	109.5
C6—C5—C10	112.2 (3)	C10—C19—H19A	109.5
C4—C5—H5	107.1	C10—C19—H19B	109.5
C6—C5—H5	107.1	H19A—C19—H19B	109.5
C10—C5—H5	107.1	C10—C19—H19C	109.5
C7—C6—C5	113.3 (3)	H19A—C19—H19C	109.5
C7—C6—H6A	108.9	H19B—C19—H19C	109.5
C5—C6—H6A	108.9	C17—C20—C21	112.4 (4)
C7—C6—H6B	108.9	C17—C20—C22	110.6 (3)
C5—C6—H6B	108.9	C21—C20—C22	110.3 (4)
H6A—C6—H6B	107.7	C17—C20—H20	107.8
C8—C7—C6	111.7 (3)	C21—C20—H20	107.8
C8—C7—H7A	109.3	C22—C20—H20	107.8
C6—C7—H7A	109.3	C20—C21—H21A	109.5
C8—C7—H7B	109.3	C20—C21—H21B	109.5
C6—C7—H7B	109.3	H21A—C21—H21B	109.5
H7A—C7—H7B	107.9	C20—C21—H21C	109.5
C14—C8—C7	112.2 (3)	H21A—C21—H21C	109.5
C14—C8—C9	109.5 (3)	H21B—C21—H21C	109.5
C7—C8—C9	110.0 (3)	C23—C22—C20	112.8 (3)
C14—C8—H8	108.4	C23—C22—H22A	109.0
C7—C8—H8	108.4	C20—C22—H22A	109.0
C9—C8—H8	108.4	C23—C22—H22B	109.0
C11—C9—C8	112.8 (3)	C20—C22—H22B	109.0
C11—C9—C10	112.9 (3)	H22A—C22—H22B	107.8
C8—C9—C10	111.4 (3)	C24—C23—C22	111.7 (3)
C11—C9—H9	106.4	C24—C23—H23A	109.3
C8—C9—H9	106.4	C22—C23—H23A	109.3
C10—C9—H9	106.4	C24—C23—H23B	109.3
C19—C10—C1	106.6 (3)	C22—C23—H23B	109.3
C19—C10—C5	109.6 (3)	H23A—C23—H23B	107.9
C1—C10—C5	107.7 (3)	O24—C24—N24	122.3 (4)
C19—C10—C9	111.5 (3)	O24—C24—C23	121.1 (4)
C1—C10—C9	111.9 (3)	N24—C24—C23	116.6 (4)
C5—C10—C9	109.4 (3)	N24—C25—C26	112.6 (4)
C9—C11—C12	113.9 (3)	N24—C25—H25A	109.1
C9—C11—H11A	108.8	C26—C25—H25A	109.1
C12—C11—H11A	108.8	N24—C25—H25B	109.1
C9—C11—H11B	108.8	C26—C25—H25B	109.1
C12—C11—H11B	108.8	H25A—C25—H25B	107.8
H11A—C11—H11B	107.7	C27—C26—C25	113.7 (4)
C13—C12—C11	111.5 (3)	C27—C26—H26A	108.8
C13—C12—H12A	109.3	C25—C26—H26A	108.8
C11—C12—H12A	109.3	C27—C26—H26B	108.8
C13—C12—H12B	109.3	C25—C26—H26B	108.8
C11—C12—H12B	109.3	H26A—C26—H26B	107.7
H12A—C12—H12B	108.0	O27—C27—C26	112.9 (3)
C12—C13—C18	111.1 (3)	O27—C27—H27A	109.0
C12—C13—C14	106.5 (3)	C26—C27—H27A	109.0
C18—C13—C14	111.9 (3)	O27—C27—H27B	109.0
C12—C13—C17	116.5 (3)	C26—C27—H27B	109.0
C18—C13—C17	110.1 (3)	H27A—C27—H27B	107.8
			
C10—C1—C2—C3	58.8 (4)	C11—C12—C13—C17	168.6 (3)
C1—C2—C3—O3	−176.7 (3)	C7—C8—C14—C15	−55.8 (5)
C1—C2—C3—C4	−56.7 (4)	C9—C8—C14—C15	−178.2 (3)
O3—C3—C4—C5	179.5 (3)	C7—C8—C14—C13	−178.8 (3)
C2—C3—C4—C5	55.9 (4)	C9—C8—C14—C13	58.7 (4)
C3—C4—C5—C6	−179.8 (3)	C12—C13—C14—C15	166.7 (3)
C3—C4—C5—C10	−53.6 (4)	C18—C13—C14—C15	−71.8 (4)
C4—C5—C6—C7	75.0 (4)	C17—C13—C14—C15	44.8 (4)
C10—C5—C6—C7	−52.1 (5)	C12—C13—C14—C8	−63.2 (4)
C5—C6—C7—C8	53.0 (5)	C18—C13—C14—C8	58.4 (4)
C6—C7—C8—C14	−177.8 (3)	C17—C13—C14—C8	175.0 (3)
C6—C7—C8—C9	−55.6 (4)	C8—C14—C15—C16	−161.2 (3)
C14—C8—C9—C11	−49.3 (4)	C13—C14—C15—C16	−34.1 (4)
C7—C8—C9—C11	−173.0 (4)	C14—C15—C16—C17	9.7 (4)
C14—C8—C9—C10	−177.4 (3)	C15—C16—C17—C20	146.1 (3)
C7—C8—C9—C10	58.9 (4)	C15—C16—C17—C13	17.9 (4)
C2—C1—C10—C19	−171.7 (3)	C12—C13—C17—C20	82.7 (4)
C2—C1—C10—C5	−54.1 (4)	C18—C13—C17—C20	−44.9 (5)
C2—C1—C10—C9	66.2 (4)	C14—C13—C17—C20	−162.8 (3)
C4—C5—C10—C19	165.9 (3)	C12—C13—C17—C16	−152.0 (4)
C6—C5—C10—C19	−69.2 (4)	C18—C13—C17—C16	80.4 (4)
C4—C5—C10—C1	50.3 (4)	C14—C13—C17—C16	−37.6 (4)
C6—C5—C10—C1	175.2 (3)	C16—C17—C20—C21	178.8 (4)
C4—C5—C10—C9	−71.6 (4)	C13—C17—C20—C21	−60.1 (5)
C6—C5—C10—C9	53.3 (4)	C16—C17—C20—C22	54.9 (5)
C11—C9—C10—C19	−64.0 (5)	C13—C17—C20—C22	176.0 (3)
C8—C9—C10—C19	64.1 (5)	C17—C20—C22—C23	−172.3 (3)
C11—C9—C10—C1	55.3 (4)	C21—C20—C22—C23	62.6 (5)
C8—C9—C10—C1	−176.6 (3)	C20—C22—C23—C24	177.6 (4)
C11—C9—C10—C5	174.6 (3)	C25—N24—C24—O24	6.6 (6)
C8—C9—C10—C5	−57.4 (4)	C25—N24—C24—C23	−173.3 (3)
C8—C9—C11—C12	47.6 (4)	C22—C23—C24—O24	−60.4 (5)
C10—C9—C11—C12	174.9 (3)	C22—C23—C24—N24	119.5 (4)
C9—C11—C12—C13	−53.0 (5)	C24—N24—C25—C26	−97.7 (5)
C11—C12—C13—C18	−64.3 (4)	N24—C25—C26—C27	59.3 (5)
C11—C12—C13—C14	57.9 (4)	C25—C26—C27—O27	55.1 (5)

### Hydrogen-bond geometry (Å, °)

**Table:**

*D*—H···*A*	*D*—H	H···*A*	*D*···*A*	*D*—H···*A*
O27—H27O···O24	0.86 (4)	1.98 (2)	2.810 (4)	164 (5)
O3—H3O···O24^i^	0.84 (2)	2.05 (2)	2.880 (5)	171 (5)
N24—H24···O3^i^	0.89 (2)	2.20 (3)	3.032 (5)	155 (4)

Symmetry codes: (i) −*x*+1, *y*+1/2, −*z*.
